# MBD2 regulates differentiation and function of Th17 cells in neutrophils- dominant asthma via HIF-1α

**DOI:** 10.1186/s12950-018-0191-x

**Published:** 2018-08-20

**Authors:** Li Xu, Wen J. Sun, Ai J. Jia, Lu L. Qiu, Bing Xiao, Lin Mu, Jian M. Li, Xiu F. Zhang, Yan Wei, Cong Peng, Dong S. Zhang, Xu D. Xiang

**Affiliations:** 1 Department of the Second Thoracic Medicine, The Affiliated Cancer Hospital of Xiangya School of Medicine and Hunan Cancer Hospital, Central South University, 283 Tongzipo Road, Changsha, 410006 Hunan China; 2 Department of Respiratory Medicine, Hunan Centre for Evidence-based Medicine, Research Unit of Respiratory Diseases, The Second Xiangya Hospital, Central South University, 139 Middle Renmin Road, Changsha, 410011 Hunan China; 3 Department of Emergency, The Second Xiangya Hospital, Central South University, 139 Middle Renmin Road, Changsha, Hunan 410011 People’s Republic of China; 4 Department of Respiratory Medicine, Peace Hospital, Changzhi Medical College, Changzhi, 046000 Shanxi China; 5 Department of Respiratory Medicine, Hunan Provincial People’s Hospital, 61 West Jiefang Road, Changsha, 410005 Hunan China; 6 Department of Respiratory Medicine, The Second Hospital, University of South China, 30 Jiefang Road, Hengyang, 421001 Hunan China; 7 Department of Respiratory, The First Hospital of Guangyuan City, 490 Juguo Road, Guangyuan, 628000 Sichuan China; 8 Dermatology and Venereology Department, Xiangya Hospital, Central South University, 87 Xiangya Road, Changsha, 410008 Hunan China

**Keywords:** Neutrophils-dominant asthma, Hypoxia inducible factor-1α, Methtyl-CpG binding domain protein 2, T helper 17 cells

## Abstract

**Background:**

T helper 17 (Th17) cells have proven to be crucial in the pathogenesis of neutrophils-dominant asthma. Hypoxia inducible factor-1α (HIF-1α) is involved in allergic responses in asthma. Our previous studies indicated that Methtyl-CpG binding domain protein 2 (MBD2) expression was increased in asthma patients. The aim of the present study is to understand how MBD2 interacts with HIF-1α to regulate Th17 cell differentiation and IL-17 expression in neutrophils-dominant asthma.

**Methods:**

A neutrophils-dominant asthma mouse model was established using female C57BL/6 mice to investigate Th17 cell differentiation and MBD2 and HIF-1α expression regulation using flow cytometry, western blot or qRT-PCR. MBD2 and HIF-1α genes were silenced or overexpressed through lentiviral transduction to explore the roles of MBD2 in Th17 cell differentiation and IL-17 release in neutrophils-dominant asthma.

**Results:**

A neutrophilic inflammatory asthma phenotype model was established successfully. This was characterized by airway hyperresponsiveness (AHR), increased BALF neutrophil granulocytes, activated Th17 cell differentiation, and high IL-17 levels. MBD2 and HIF-1α expression were significantly increased in the lung and spleen cells of mice with neutrophils-dominant asthma. Through overexpression or silencing of MBD2 and HIF-1α genes, we have concluded that MBD2 and HIF-1α regulate Th17 cell differentiation and IL-17 secretion. Moreover, MBD2 was also found to regulate HIF-1α expression.

**Conclusions:**

Our findings have uncovered new roles for MBD2 and HIF-1α, and provide novel insights into the epigenetic regulation of neutrophils-dominant asthma.

**Electronic supplementary material:**

The online version of this article (10.1186/s12950-018-0191-x) contains supplementary material, which is available to authorized users.

## Background

Classic eosinophilic allergic asthma is characterized by a Th2 immune response as well as IL-4 and IL-5 production [1]. A meta-analysis of clinical samples found that eosinophil inflammation was the most prevalent subtype of asthma. The airway cells in nearly 50% of asthmatic patients were not solely eosinophils, but a combination of two kinds of cells, eosinophils and neutrophils, which infiltrated airway inflammation and were associated with poor corticosteroid response and severe asthma (which we have called neutrophils-dominant asthma) [2–6].

Allergens play an important role in asthma, but environmental factors, such as infection and chemical factors, also contribute to the exacerbation of asthma via a Th17-type immune response. Th17 cells are initially composed of CD4^+^ T cells. Retinoid-related orphan nuclear receptor γt (RORγt) is a key transcriptional regulator of Th17 cell differentiation. IL-17 produced by Th17 cells can recruit neutrophils to the airway and is less susceptible to inhibition by glucocorticoids than IL-4 and IL-5 produced by Th2 cells [7]. Numerous reports suggest that increased expression of IL-17 is associated with neutrophils-dominant asthma [8]. Therefore, we sought to establish a neutrophil-predominant inflammatory phenotype asthma model. We used 100 μg of HDM and OVA combined with 15 μg of LPS to establish a neutrophil-predominant asthma model and to further study the relationship between the changes of Th17 cells and the epigenetic alterations.

About 90% of severe asthma attacks feature hypoxia, which exacerbates the condition. The effect of hypoxia is regulated via a specific transcription factor, hypoxia inducible factor-1 (HIF-1), which is a heterodimer consisting of HIF-1α and -1β subunits [9]. Studies have shown that HIF-1α deficiency diminishes Th17 cell development but enhances Treg cell differentiation and protects mice from autoimmune neuro-inflammation [10]. In an allergic airway inflammation model, hypoxia was found to increase airway inflammation, but HIF-1α knockout mice were resistant to airway inflammation [11]. Therefore, it is logical to hypothesize that HIF-1α may be involved in the pathogenesis of neutrophils-dominant asthma by regulating differentiation of Th17 cells.

Genetic and environmental factors also contribute to the development of asthma. For instance, DNA methylation is involved in CD4^+^ T cells’ differentiation into T effector cells. Recent studies suggest that DNA methylation has an environmental impact on different types of “imprinting.” A DNA methylation imprint can be “read” by a methylated-CpG binding domain (methyl-CpG binding domain proteins, MBDs) conservative family [12–14]. MBD2, specifically, can bind to the promoter region of a target gene and change in the post-transcriptional modification of histones through the recruitment of other molecules, therefore changing the chromatin structure and regulating the expression of target genes [12–14]. Our previous work indicates that, compared to healthy volunteers, MBD2 and HIF-1α expression in CD4^+^ T cells was increased in the peripheral blood of patients with asthma. What’s more, expression of HIF-1α decreased significantly in MBD2 knockout Jurkat T cells. MBD2 expression was also detected in splenic CD4^+^ T cells and increased after differentiation stimulation. However, other MBD family members were not detected in splenic CD4^+^ T cells. Compared to wild-type mice, splenic CD4^+^ T cell differentiation was reduced in MBD2^−/−^ mice, as was Th17’s production of IL-17. Therefore, MBD2 may have a close relationship with the immunological pathogenesis of asthma and contribute to Th17 cell differentiation and IL-17 expression through HIF-1α. Understanding the role of MBD2 and HIF-1α in neutrophils-dominant asthma may offer a theoretical basis for treatment.

## Methods

### Asthma mouse model

Female C57BL/6 mice (6–7 weeks old, 18–20 g) were provided by The Second Xiangya Hospital Animals Center (Changsha, China) and maintained under specific pathogen-free conditions. All experimental procedures were approved by the Animal Care and Use Committee of Central South University. On days 0, 1, and 2, mice (*n* = 6/group) in the neutrophils-dominant asthma group were given an intraperitoneal sensitization injection containing 100 μg of HDM (10 mg/ml, house dust mice, Greer Laboratories, Lenoir, N.C., USA), 100 μg of OVA (1 mg/ml, Ovalbumin, Grade V, Sigma Aldrich) and 15 μg of LPS (1 mg/ml, lipopolysaccharide, Sigma) with 2 mg of aluminum hydroxide (Sigma) dissolved in 200 μl of saline [15]. On days 14, 15, 18, and 19, the mice were challenged with atomized 6% OVA solution for 30 min before 100 μg / 10 μl HDM was applied intranasally. A saline control group was sensitized and boosted with saline only, but the injection location, time, and dose were consistent with those of neutrophils-dominant asthma group.

Mice (n = 6/group) in the conventional asthma group were given an intraperitoneal sensitization injection with 25 μg of OVA (1 mg/ml) and 1 mg of aluminum hydroxide on days 0 and 7, and then challenged with an atomized 6% OVA solution excitation for 30 min on days 14, 15, 16, 17, 18, 19, and 20 [16]. All mice were killed on day 21 for analysis.

### Assessment of AHR

Methacholine (Mch)-induced airway resistance was measured on day 21 by direct plethysmography (Buxco Electronics, RC System, Wilmington, NC, USA), according to published methods [17]. Mice were anesthetized, tracheotomized, and then intubated. First, baseline lung resistance (RL) was measured for 1 min. Then, mice were given 10 μl of atomized saline and 10 μl of Mch at increasing doses (0.39 mg/ml, 0.78 mg/ml, 1.56 mg/ml, 3.12 mg/ml) to stimulate the airway and RL was recorded once again.

### BALF processing

Bronchoalveolar lavage fluid (BALF) was collected after three injections of 0.5 ml of saline (37 °C) through a tracheal cannula into the lung. BALF cells were centrifuged and resuspended in cold PBS. BALF cells were counted using a counting chamber. For differential BALF cell counts, cytospin preparations were made (1500 rpm, 5 min, 4 °C, Eppendorf Centrifuge Configurator, Hamburg, Germany). Next, cells were fixed and stained with Wright-Giemsa stain and 200 cells were counted under a light microscope.

### Histopathology

Lungs were first fixed with 10% formalin via the trachea, and then removed and stored in 10% formalin. Fixed lung tissues were paraffinized and sectioned (5 μm) for hematoxylin and eosin (H&E) staining. Lung tissues were stained for immunohistochemistry (neutrophil-specific antibody [0.2 mg/ml, anti-Gr1, Biolegend, San Diego, CA, USA], eosinophil antibody [1 mg/ml, anti-ECP, Biorbyt, Cambridge, United Kingdom], MBD2 antibody [Abcam, Cambridge, United Kingdom] and HIF-1α antibody [Proteintech]). Select stained sections from each group were collected and assessed for neutrophil, eosinophil, MBD2, and HIF-1α protein expression.

### Bronchial lung tissue suspensions

Mouse bronchial lung fluid was collected, washed once with 5X antibiotic, and washed twice in PBS. Then, lungs were digested with collagenase1 (0.5 mg/ml, Sigma) and 10 μg/ml DNase in RPMI medium for 1 h in a 37 °C water bath using a shaking incubator. After digestion, tissue was filtered using a 70 μm Cell Strainer (BD Falcon, New York, USA) and cells were centrifuged and resuspended in a 10% FBS culture medium.

### T cell purification, activation, and staining

Splenic CD4^+^ T cells from asthma model mice were selected using microbead (130–049-201, Miltenyi Biotec, Germany) sorting and were seeded in 12-well flat bottom plates for 24 h. Five hours later, cells were restimulated with 50 ng/ml of phorbol-12-myristate-13-acetate (PMA) (Multi Sciences Company, China), 1 μg/ml of ionomycin (Multi Sciences Company), and 3 μg/ml of monensin (Multi Sciences Company) for 5 h. Lung cells were then stained for surface marker FITC-anti CD4^+^ cytokine antibody (Biolegend) followed by fixation and permeabilization using fixation and permeabilization buffers (Multi Sciences Company) for 15 min. After washing with permeabilization buffer, lung and splenic CD4^+^ T cells were stained with intracellular markers APC-anti-IL-17 and PE-anti-IL-4 cytokine antibodies (Biolegend) in a permeabilization buffer for 20 min. Isotype controls were employed in the control group. Flow cytometry was performed and data were analyzed using FACSCalibur and FlowJo version X software.

### Th17 cell differentiation

Splenic naïve CD4^+^ T cells were purified from mouse spleens via magnetic isolation (Miltenyi Biotec, Bergisch Gladbach, Germany). For the preparation of spleen cell suspensions, spleens from 8-week-old female C57BL/6 mice were removed and minced using a nylon mesh (70 μm pore size). After the cells were pelleted, erythrocytes were lysed with a hypotonic buffer (0.15 mM NH_4_Cl, 10 mM KHCO_3_, 0.1 mM Na_2_EDTA). Cells were washed in PBS and incubated with anti-CD4 antibody for 15 min at 4 °C. Cells were then conducted onto a magnetic separator to isolate CD4^+^ T cells and were collected with positive selection. For Th17 cell directed differentiation, 1 × 10^5^ naïve CD4^+^ T cells from C57BL/6 mice were activated and cultured for 6 days with anti-CD3/anti-CD28 mouse Dynabeads (Invitrogen) under Th17 cell polarizing conditions in the presence of 5 ng/ml TGF-beta, 20 ng/ml IL-1beta, 20 ng/ml IL-6, 10 ng/ml IL-21, 10 μ g/ml anti-IL-4 and 10 μg/ml anti-INF-gamma [18, 19]. All cytokines were purchased from Biolegend Co.

### Lentiviral siRNA transduction ex vivo

Splenic CD4^+^ T cells collected from normal mice were plated on a 12-well plate at a concentration of 1 × 10^5^ cells per well. The cells were cultured in 1640 medium without serum. After 24 h, the medium was carefully removed. Cells were placed in 1640 culture with no serum after suspension (2 × 10^6^/tube) and virally transfected at MOI = 20 (using the appropriate amount of virus) in the presence of polybrene. These cells were then seeded in 12-well flat bottom plates. Splenic CD4^+^ T cells were transfected with chemosynthesis MBD2 siRNA sequence (S) 5’-GTTTGGCTTAACACATCTCAA-3′; HIF-1α siRNA sequence (S) 5’-GCCACTTTGAATCAAAGAAAT-3′. The virus culture medium was removed by centrifugation after 4 h of transfection and replaced with 1640 complete medium with Th17 cell differentiation conditions. After 3 days, the cells were collected for detection.

### Western blot

Total proteins were prepared using RIPA lysis buffer supplemented with protease inhibitors. Western blot was carried out by probing membranes with the indicated primary antibodies followed by incubation with an HRP-conjugated secondary antibody. MBD2 and HIF-1α antibodies were applied in the same manner.

### qRT-PCR

Lungs were harvested from mice for qRT-PCR (Quantitative Reverse Transcription PCR) analysis according to published methods [20].

### Statistical analysis

All data are expressed as means ± SEM unless otherwise specified and all in vitro experiments were conducted with three independent replications (*p* < 0.05 was considered statistically significant). SPSS version 17.0 was used for statistical analysis using one-way ANOVA and Bonferroni’s post hoc tests where appropriate.

## Results

### A neutrophils-dominant asthma mouse model was established

**Fig. 1:**
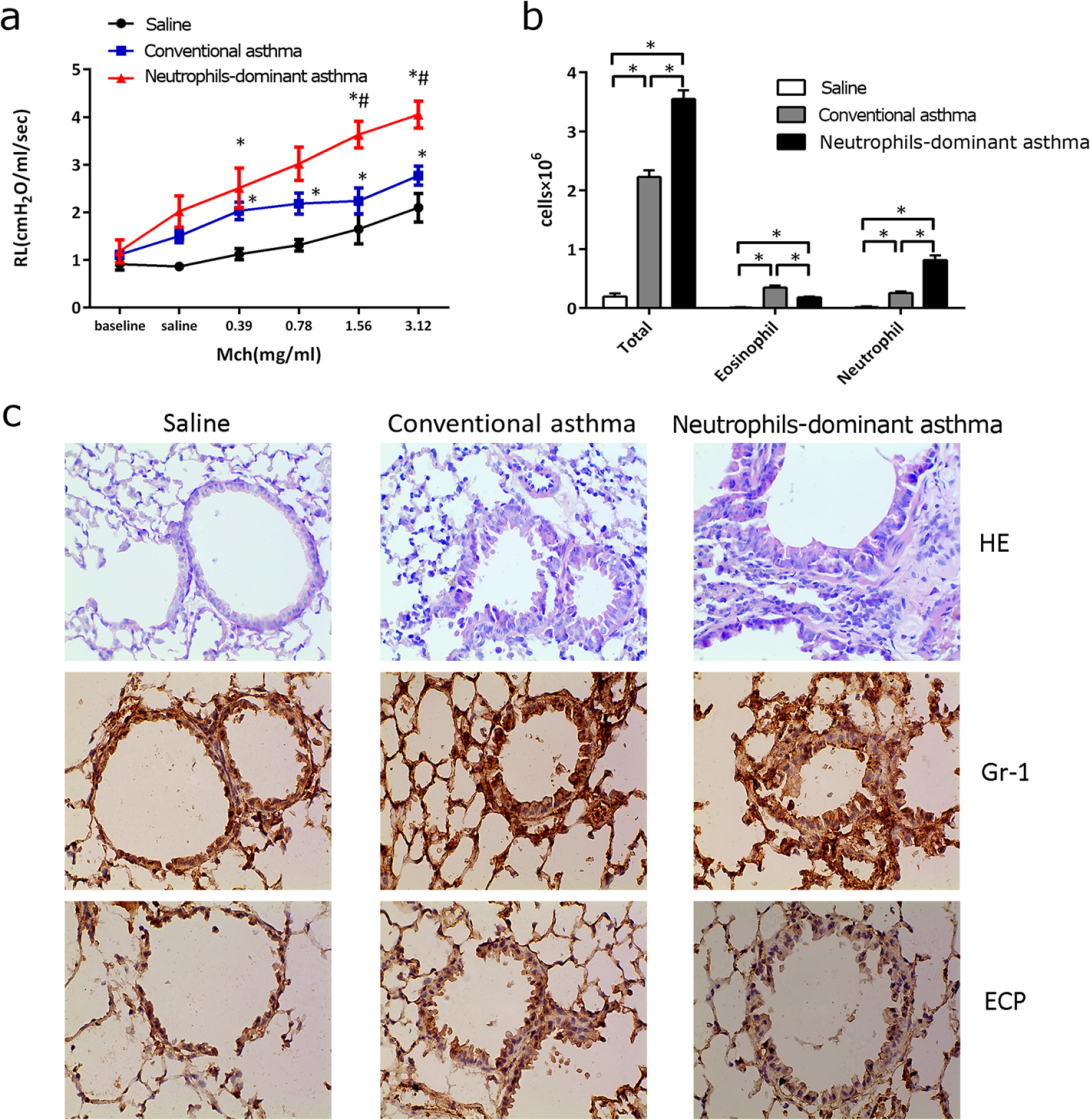
Establishment of a neutrophils-dominant asthma mouse model **a** Pulmonary resistance in control, conventional asthma, and neutrophils-dominant asthma groups, *p < 0.05 compared to control. #*p* < 0.05 compared with conventional asthma. **b** Total neutrophil and eosinophil cells in BALF from all groups. *p < 0.05 compared to other group. **c** Lung tissues stained with H&E and for neutrophil-specific antibody (anti-Gr1), eosinophil antibody (anti-ECP) in all groups. One-way ANOVA with Bonferroni’s post hoc tests were applied to analyze the results for significant differences (*p < 0.05 or #*p* < 0.05)

We measured Mch-induced airway resistance and BALF cells on day 21. Compared to the saline and conventional asthma mice, the neutrophils-dominant asthma mice had greater baseline pulmonary resistance. After the Mch challenge these mice also had the greatest increase in resistance (Fig. 1a). Compared to the saline and conventional asthma mice, the neutrophils-dominant asthma mice had the most BALF cells (Fig. 1b), most inflammatory cells infiltrated around the bronchi and mucus excretion in the airway lumen (Fig. 1c). Differential BALF cell counts and Gr-1 (neutrophil-specific antibody) immunohistochemistry confirmed a more significant influx of neutrophils (but not eosinophils) into the lung in the neutrophils-dominant asthma group compared to the conventional asthma group (Fig. 1b, c). Eosinophil cell counts were most in conventional asthma group. ECP (eosinophil-specific antibody) immunohistochemistry indicated that, in both the neutrophils-dominant and conventional asthma groups, eosinophil infiltration was present and was greater than that observed in the saline group, and the neutrophils-dominant asthma group increased more, but not significantly from the conventional asthma animals.

### Neutrophils-dominant asthma mediated by Th17 cells

**Fig. 2:**
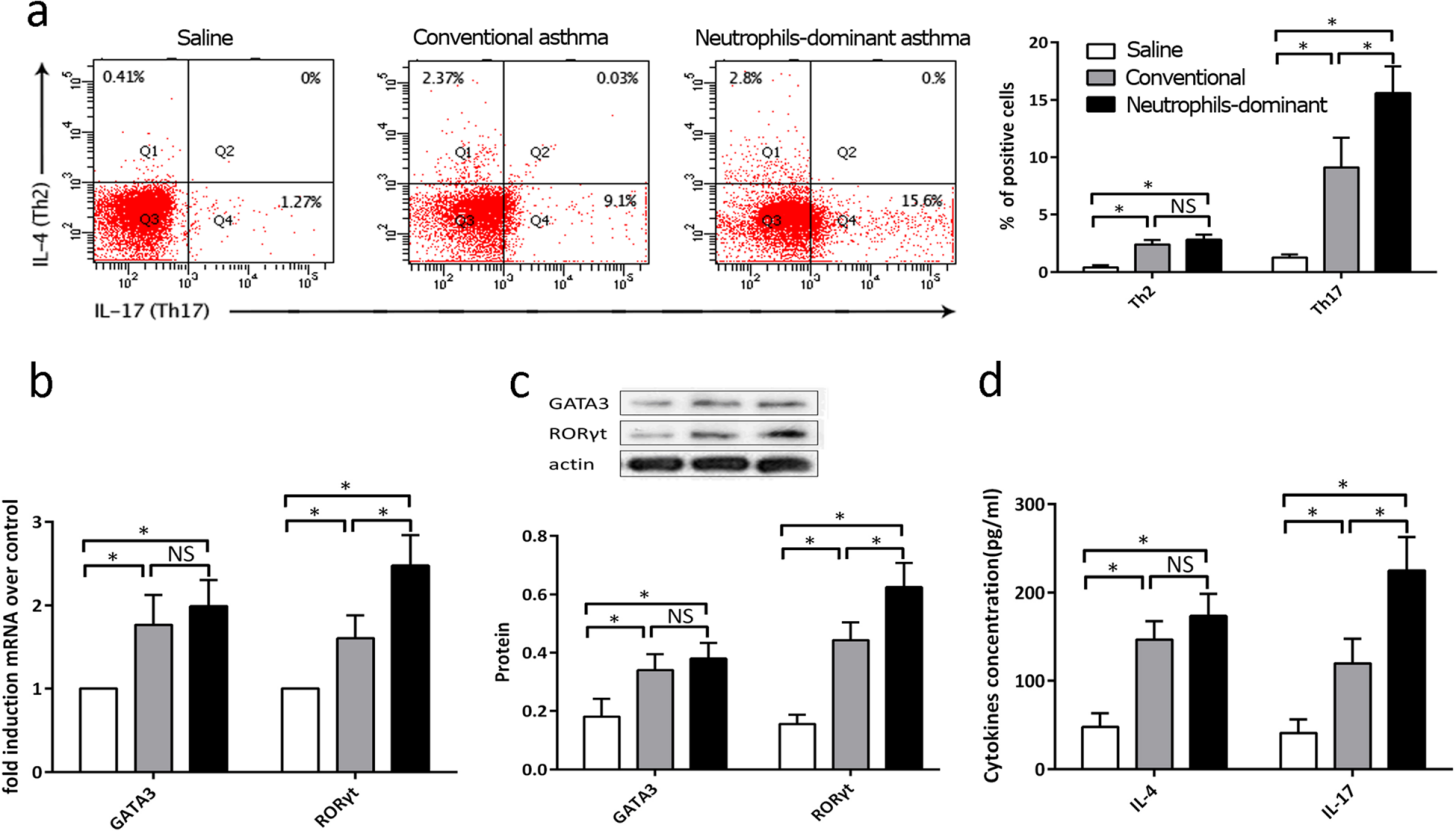
Neutrophils-dominant asthma mediated by Th17 cells **a** Th17 and Th2 cells in splenocytes, stained for intracellular APC-anti-IL-17 and PE-anti-IL-4 and assessed using flow cytometry. **b** and **c** qRT-PCR and western blot used to measure RORγt and GATA3 mRNA and protein expression in all groups. **d** Secreted IL-17 and IL-4 in splenic CD4^+^ T cells using ELISA. *p < 0.05 compared to other groups. One-way ANOVA with Bonferroni’s post hoc tests were applied to analyze the results for significant differences (*p < 0.05)

IL-17 and IL-4 are representative cytokines of Th17 and Th2 cells respectively. Th17 and Th2 cells were tested in splenic CD4^+^ T cells, and the cells were then subjected to intracellular staining of APC-anti-IL-17 and PE-anti-IL-4 using flow cytometry analyses. Th17 levels were elevated in splenic CD4^+^ T cells from the neutrophils-dominant asthma group compared to the conventional asthma group, but Th2 cell levels were not significantly different between the two groups (Fig. 2a). Results of qRT-PCR and western blot analyses showed a similar tendency to the results found using flow cytometry. RORγt, the key transcriptional regulator of Th17 cells, was highly expressed in the neutrophils-dominant asthma group compared to the conventional asthma group, whereas GATA3, the key transcriptional regulator of Th2 cells, was not overtly different between the two groups (Fig. 2b, c). Secreted IL-17 in splenic CD4^+^ T cells was significantly increased in the neutrophils-dominant asthma group compared to the conventional asthma group, but IL-4 was not obviously different in the neutrophilic group compared to the conventional group (Fig. 2d).

### Expression of MBD2 in neutrophils-dominant asthma

**Fig. 3:**
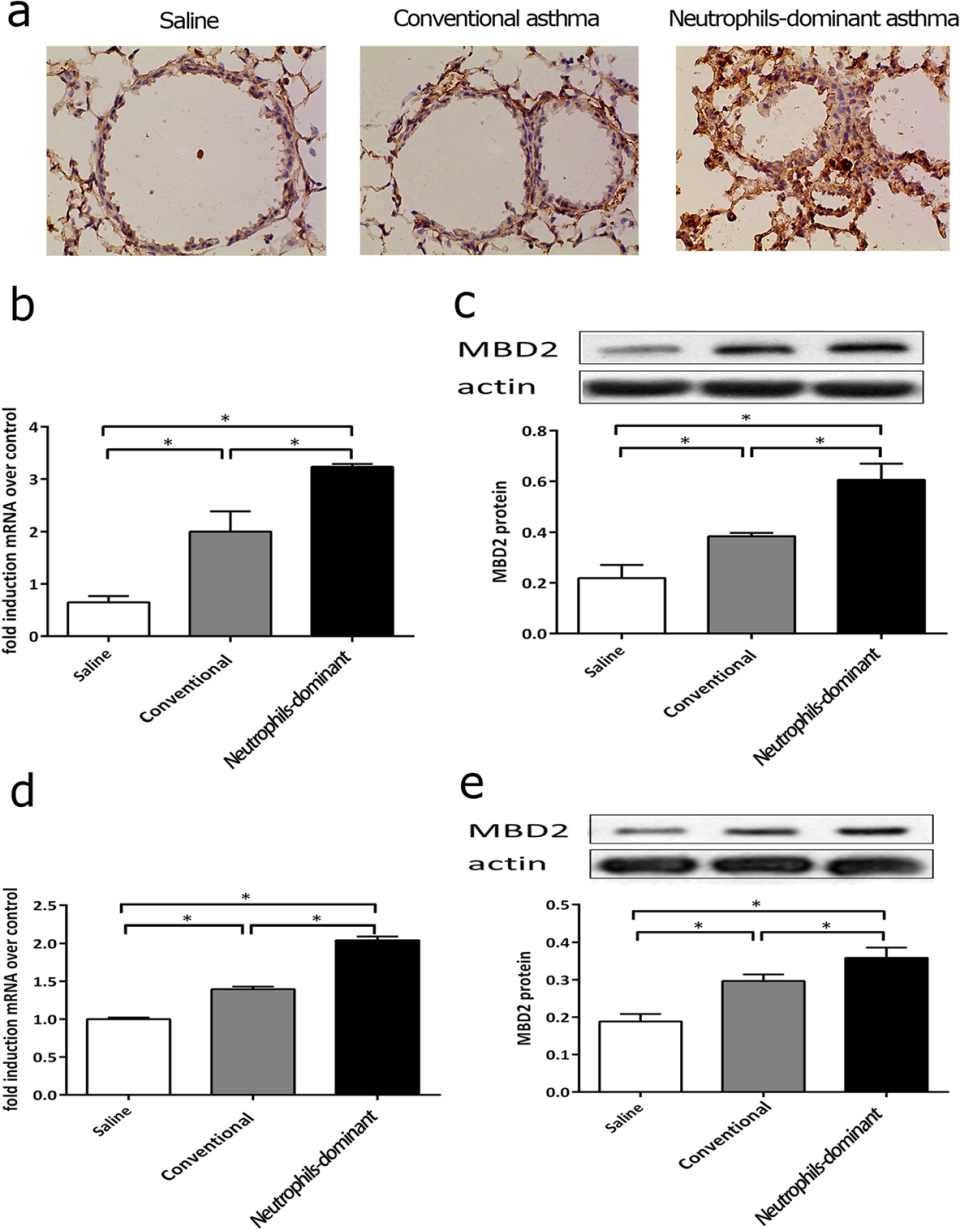
MBD2 expression in all groups **a** Lung tissues stained for anti-MBD2. **b** and **c** qRT-PCR and western blot used to measure MBD2 mRNA and protein expression in lungs. **d** and **e** qRT-PCR and western blot used to measure MBD2 mRNA and protein expression in splenocytes. *p < 0.05 compared to other groups. One-way ANOVA with Bonferroni’s post hoc tests were applied to analyze the results for significant differences (*p < 0.05)

Histological analyses of lungs from the neutrophils-dominant asthma group showed more abundant cells with MBD2 compared to the conventional asthma group (Fig. 3a). MBD2 expression in lung (Fig. 3b, c) and splenic CD4^+^ T cells (Fig. 3d, e) from the neutrophils-dominant asthma group were significantly increased compared to the conventional asthma group.

### MBD2 stimulates Th17 cell differentiation and IL-17 expression

**Fig. 4:**
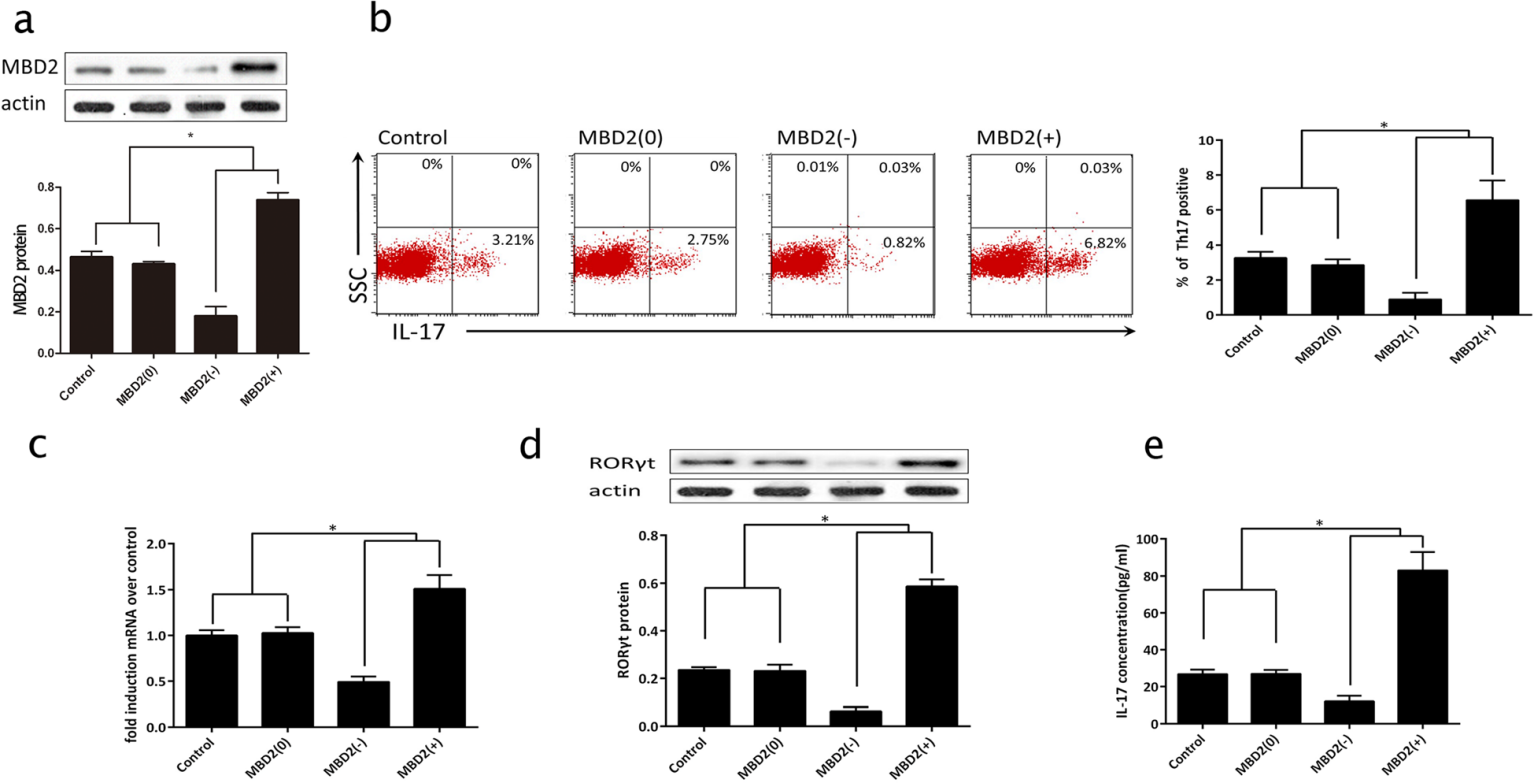
MBD2 stimulates Th17 cell differentiation and IL-17 expression **a** western blot to verify MBD2 transfection. **b** Flow cytometry used to measure the ratio of positive Th17 cells in splenic CD4^+^ T cells of all groups with MBD2 gene silencing (M[−]) or overexpression (M[+]). **c** and **d** qRT-PCR and western blot used to measure RORγt mRNA and protein expression with MBD2 gene silencing or overexpression. **e** Secreted IL-17 in splenic CD4^+^ T cells measured using ELISA with MBD2 gene silencing (M[−]) or overexpression (M[+]).*p < 0.05 compared to other groups. One-way ANOVA with Bonferroni’s post hoc tests were applied to analyze the results for significant differences (*p < 0.05)

We tested MBD2 gene silencing (MBD2[−]) or overexpression (MBD2[+]) in splenic naïve CD4^+^ T cells from normal mice, and proved that transfection was successful (Fig. 4a). The frequency of IL-17-positive cells, mRNA, and protein expression of RORγt, and IL-17 in splenic naïve CD4^+^ T cells in the MBD2(−) group were lower than those observed in a mock-transfected control group (MBD2[0]) (Fig. 4b-e) and this result was reversed in the MBD2(+) group.

### Expression of HIF-1α in neutrophils-dominant asthma

**Fig. 5:**
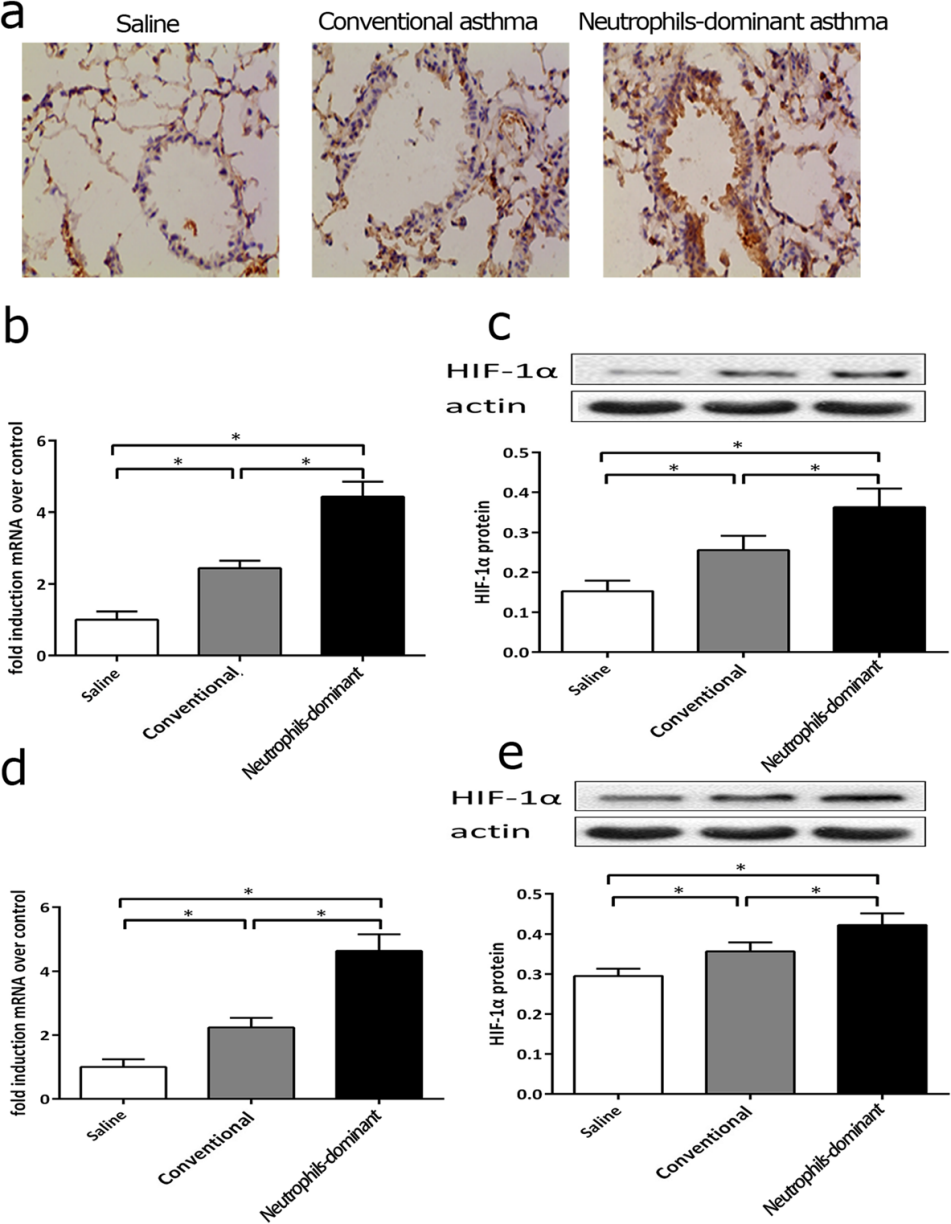
Expression of HIF-1α in all groups **a** Lung tissues were stained for anti- HIF-1α. **b** and **c** qRT-PCR and western blot analyses used to measure HIF-1α mRNA and protein expression in lung samples from all groups. **d** and **e** qRT-PCR and western blot analyses used to measure HIF-1α mRNA and protein expression in splenocytes from all groups. *p < 0.05 compared to other groups. One-way ANOVA with Bonferroni’s post hoc tests were applied to analyze the results for significant differences (*p < 0.05)

HIF-1α immunohistochemistry indicated that HIF-1α mRNA and protein expression in conventional asthmatic mouse and neutrophils-dominant asthmatic mouse lung tissues were greater than that observed in the control group (Fig. 5a), and HIF-1α expression in lung (Fig. 5b, c) and splenic naïve CD4^+^ T cells (Fig. 5d, e) from the neutrophils-dominant asthma group was greater than in the conventional asthma group.

### Correlation between HIF-1α and MBD2

**Fig. 6:**
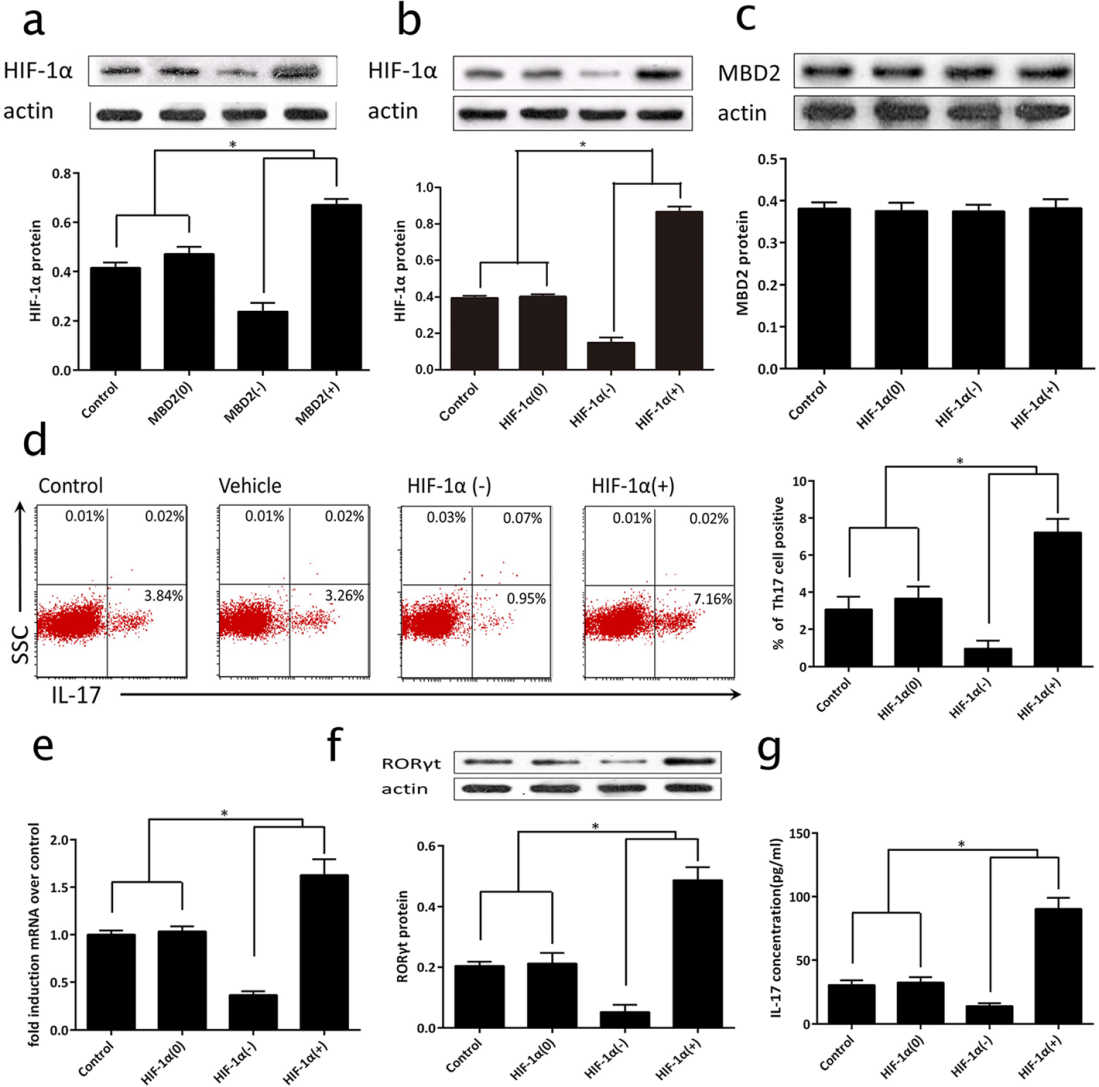
HIF-1α expression with MBD2 gene silencing or overexpression and MBD2 expression with HIF-1α gene silencing or overexpression; HIF-1α stimulates Th17 cell differentiation and IL-17 expression **a** western blot to measure HIF-1α protein expression in all groups with MBD2 gene silencing (M[−]) or overexpression (M[+]). **b** western blot to verify HIF-1α transfection. **c** western blot to measure MBD2 protein expression in all groups with HIF-1α gene silencing (H[−]) or overexpression (H[+]). **d** Flow cytometry used to measure the ratio of positive Th17 cells in splenic CD4^+^ T cells of all groups with HIF-1α gene silencing or overexpression. **e** and **f** qRT-PCR and western blot used to measure RORγt mRNA and protein expression in all groups with HIF-1α gene silencing or overexpression. **g** Secreted IL-17 in splenic CD4^+^ T cells as measured using ELISA with HIF-1α gene silencing or overexpression. *p < 0.05 compared to other groups. One-way ANOVA with Bonferroni’s post hoc tests were applied to analyze the results for significant differences (*p < 0.05)

To illustrate the correlation between HIF-1α and MBD2, we first silenced or overexpressed the MBD2 gene in mouse splenic CD4^+^ T cells. HIF-1α protein expression in MBD2-silenced (MBD2[−]) CD4^+^ T cells was lower than in the mock-transfected group (MBD2[0]) (Fig. 6a). HIF-1α mRNA and protein expression from MBD2 overexpressed (MBD2[+]) mouse splenic naïve CD4^+^ T cells were observed to be greater than in the MBD2[0] group (Fig. 6a). Next, we silenced or overexpressed the HIF-1α gene in mouse splenic CD4^+^ T cells, and proved that transfection was successful (Fig. 6b). However, there was no statistically significant difference in MBD2 protein expression between the groups (Fig. 6c). These data suggest that MBD2 is in the upstream and regulates HIF-1α expression.

### Ratios of positive Th17 cell, RORγt expression, and IL-17 production with HIF-1α gene silencing or overexpression

HIF-1α gene silencing (HIF-1α[−]) or overexpression (HIF-1α[+]) in splenic CD4^+^ T cells was confirmed and the frequency of IL-17-positive cells, mRNA and protein expression of RORγt, and IL-17 in mouse splenic naïve CD4^+^ T cells in the HIF-1α(−) group were lower than in the mock-transfected group (HIF-1α[0]) (Fig. 6d-g). This result was reversed in the HIF-1α(+) group.

### The frequency of IL-17-positive cells, RORγt expression, and IL-17 production with MBD2 and HIF-1α gene silencing or overexpression

**Fig. 7:**
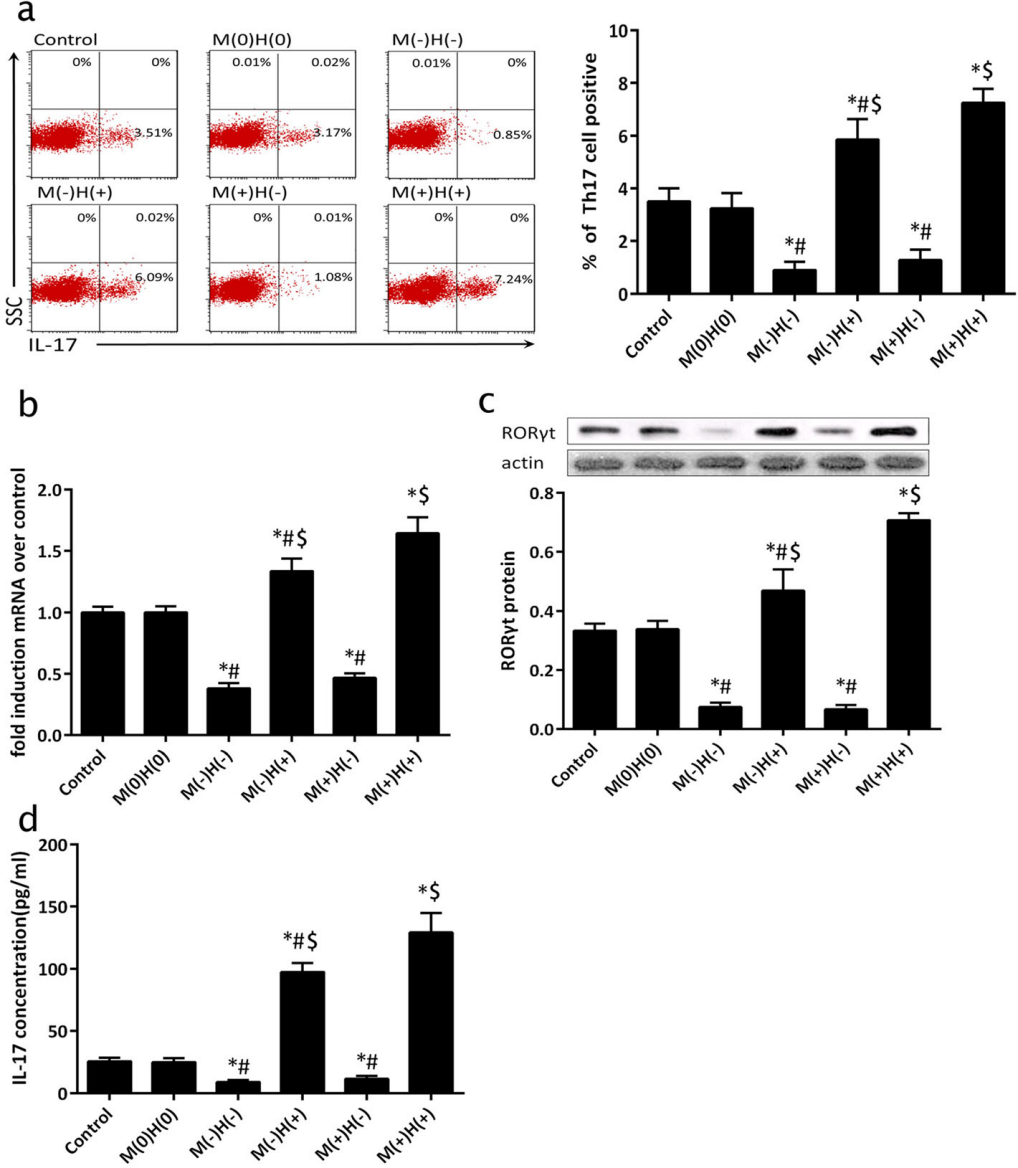
Ratio of positive Th17 cells, RORγt expression, and IL-17 expression with MBD2 and HIF-1α gene silencing or overexpression **a** Flow cytometry used to measure the ratio of positive Th17 cells in splenic CD4^+^ T cells of all groups with MBD2/HIF-1α gene silencing or overexpression. **b** and **c** qRT-PCR and western blot used to measure RORγt mRNA and protein expression in all groups with MBD2 and HIF-1α gene silencing or overexpression. **d** Secreted IL-17 in splenic CD4^+^ T cells as measured using ELISA with MBD2 and HIF-1α gene silencing or overexpression. *p < 0.05 compared to control. #*p* < 0.05 compared with the M(+)H(+) group. $*p* < 0.05 compared with the M(−)H(−) group. One-way ANOVA with Bonferroni’s post hoc tests were applied to analyze the results for significant differences (*p < 0.05 or #*p* < 0.05 or $*p* < 0.05)

The frequency of IL-17-positive cells, RORγt protein expression, and IL-17 in mouse splenic naïve CD4^+^ T cells were observed to be the highest in the M(+)H(+) group (MBD2 gene and HIF-1α co-overexpression) and were observed to be greater than in the M(+)H(−) group (MBD2 overexpression and HIF-1α silencing) (*p* < 0.05), the M(−)H(+) group (MBD2 silencing and HIF-1α overexpression), the M(−)H(−) group (MBD2 and HIF-1α co-silencing), or the M(0)H(0) group (mock-transfected group) (Fig. 7a-d). This result indicates that co-overexpression of MBD2 and HIF-1α (M[+]H[+]) promotes Th17 cell differentiation and IL-17 expression to the greatest extent.

The frequency of IL-17-positive cells, RORγt protein expression, and IL-17 in mouse splenic CD4^+^ T cells were significantly lower in the M(−)H(−) group (MBD2 and HIF-1α co-silencing), compared to the M(−)H(+) group (*p* < 0.05), the M(+)H(+) group, and the M(0)H(0) group. There was no statistically significant difference between the M(+)H(−) and M(−)H(−) groups (*p* > 0.05; Fig. 7a-d). These results indicate that MBD2 and HIF-1α co-silencing (M[−]H[−]) hinders Th17 cell differentiation and IL-17 expression. Further, HIF-1α, which is in the downstream of MBD2, regulates Th17 cell differentiation and IL-17 expression.

## Discussion

Asthma is a chronic inflammatory disorder of the airways characterized by an associated increase in airway responsiveness. Clinically, many asthma patients fail to respond satisfactorily to standard steroid therapy. This type of steroid-resistant severe asthma has been previously linked to the presence of neutrophilic inflammation in the lungs [21, 22]. In order to better study the mechanisms of neutrophilic asthma, it is necessary to build a neutrophil-predominant inflammatory asthma model.

OVA is a classic allergen for inducing asthma. Epithelial damage by HDM allergens leaves the tissue more vulnerable during subsequent exposure to allergens [23] and air toxins (biotoxins) such as endotoxin (LPS). LPS is used as an adjuvant in asthma models and associated with asthma severity as well as the occurrence of asthma exacerbation [24, 25]. Increasing LPS doses shifted the predominant eosinophilic lung inflammation induced by HDM to a neutrophil-dominated inflammation, reproducing conditions of mixed eosinophilic and neutrophilic infiltrations within the airways of some patients with asthma [15]. In murine asthma models, LPS or HDM administration via the airways increased Th17 cell response and the production of IL-17 [15]. In a previous study, we showed that 100 μg HDM + 100 μg OVA + 15 μg LPS was successful for establishing a neutrophils-dominant asthma [26].

In the present study, mice with neutrophils-dominant asthma had more AHR, the highest total BALF cell and lung neutrophil counts, and more lung inflammatory cells and neutrophil infiltration compared to mice with conventional asthma and the control group.. The spleen is the classic observation target for immune cells, and Th17 cell levels were higher in the splenocytes observed from neutrophilic asthma mice compared to conventional asthma mice. However Th2 cell levels did not differ between the two asthma groups. This shows that neutrophilic asthma mice primarily exhibited Th17 cell differentiation, and the recruitment of neutrophils, thus promoting the development of asthma. And the data in detail was published by our team [26].MBD2, a DNA methylation regulation element, can regulate T cell differentiation [27, 28]. Although little is known about epigenetic regulation in neutrophils-dominant asthma, in our study we report that MBD2 expression in splenic CD4^+^ T cells and lungs from neutrophils-dominant asthma mice was significantly higher compared to conventional asthma mice and the control group. MBD2 can also stimulate Th17 cell differentiation and IL-17 expression. Apparently, genetic abnormalities are involved in the pathogenesis of neutrophils-dominant asthma and promote Th17 cell differentiation and IL-17 expression.

HIF-1α is a crucial sensor of oxygen and is responsible for initiating cellular responses to hypoxia. Under normoxia, the HIF-1α protein degrades rapidly, while hypoxia leads to stabilization and accumulation of HIF-1α. Under certain normoxic conditions, however, HIF-1α expression can be increased [29, 30]. For example, HIF-1α expression is increased in asthma patients’ lung tissue and BALF and is also increased in patients experiencing rhinitis stimulated by antigens. HIF-1α expression is also significantly increased in mice with allergic airway inflammation [29]. Expression of HIF-1α can be detected in purely antigen stimulation-induced allergic lung inflammation due to reduced perfusion, tissue edema, vascular injury, aerobic inflammatory cell aggregation, and other inducers of allergic inflammation. So, we speculate that hypoxia is present and aggravated due to increased expression of HIF-1α [29]. Agents that inhibit HIF-1α (e.g., 2- methoxyestradiol and HIF-1 siRNA) lead to reductions in HIF-1α and VEGF expression, allergic airway response [11, 31], inflammatory cell infiltration, airway hyper-responsiveness, and Th2 cytokine levels [11]. Inhibition of HIF-1α expression can reduce airway inflammation and remodeling, suggesting that HIF-1α may be involved in the pathogenesis of asthma [29].

In Jurkat T cells, Th17 cell differentiation can be induced by HIF-1α through the HIF-1/Stat3/RORγt/IL-17 pathway [29]. ChIP analysis confirmed that HIF-1α is a transcription factor mainly due to its roles in regulating Th17 cell differentiation. HIF-1α acts as a promoter of RORγt, and directly collaborates with RORγt to activate Th17 signature genes, such as IL-17A, through mechanisms involving p300 recruitment and histone acetylation.

In our study, HIF-1α expression increased in mouse splenic CD4^+^ T cells and lung tissues in both the neutrophils-dominant asthma and conventional asthma groups, but this was more significant in the neutrophils-dominant asthma group. The results of our in vitro cell experiments show that Th17 cell differentiation and IL-17 expression increased significantly with HIF-1α gene overexpression, and decreased with HIF-1α gene silencing. This indicates that HIF-1α stimulates Th17 cell differentiation and IL-17 expression in splenic CD4^+^ T cells. As we have previously stated, about 90% of severe asthma attacks feature hypoxia which exacerbates the condition, and this hypoxic effect is regulated mainly through HIF-1. We have supposed that HIF-1 was not the initiating element in neutrophils-dominant asthma. After all, at the initiation of an asthma attack, there is no obvious hypoxia, but with asthma symptom exacerbation, severity of hypoxia increases. HIF-1α may be involved in the persistence and deterioration of neutrophils-dominant asthma through Th17 cell function and neutrophil recruitment.

Additionally, lentiviral MBD2 overexpression can promote HIF-1α expression in murine CD4^+^ T cells, suggesting that MBD2 is in the upstream of HIF-1α, and that MBD2, combined with HIF-1α and related signaling pathways, may be involved in Th17 cell differentiation and IL-17 expression.

MBD2 and HIF-1α overexpression and silencing with a lentivirus indicated that MBD2+/ HIF-1α + lentivirus co-transfection can maximize Th17 cell differentiation and IL-17 expression. MBD2-/HIF-1α + lentivirus co-transfected can partly counteract Th17 cell differentiation with double-overexpression, but this differentiation remains higher than normal, indicating that it is involved in methylation, and that MBD2 silencing can suppress the differentiation of Th17 cells. Also, silencing of MBD2 can inhibit HIF-1α downstream. This indicates that MBD2 may not be directly upstream of Th17 cell differentiation, and may play its role through HIF-1α. Regarding MBD2+/HIF-1α-, the sharp declines in Th17 cell differentiation and IL-17 expression may indicate that HIF-1α can directly regulate Th17 cell differentiation, which is consistent with previous results [10]. With double-silencing of MBD2-/HIF-1α-, Th17 cell differentiation and IL-17 expression were observed to be the lowest, but this was not statistically significant compared with MBD2+/HIF-1α-. So, likely the effect of MBD2 is indeed exerted through HIF-1α. When HIF-1α was interrupted, MBD2’s effects were completely blocked. These results indicate that MBD2 is in the upstream of HIF-1α, and its regulation of Th17 cell differentiation and IL-17 expression is mediated by HIF-1α.

We supposed that there might be some other factors involved with MBD2 and HIF-1α, one of them being insulin-like growth factor binding protein-3 (IGFBP-3). In an OVA-induced murine model of allergic asthma, IGFBP-3 expression was decreased [32], and levels of HIF-1α and HIF-2α in nuclear protein extracts from lung tissues were increased at 48 h after OVA inhalation compared to levels measured 48 h after saline inhalation [33]. Restoration of IGFBP-3 either by recombinant IGFBP-3 treatment or adenoviral IGFBP-3 gene transfer to OVA-inhalation mice substantially attenuated the increases in HIF-α activity, VEGF production, vascular leaking, and attenuated antigen-induced airway inflammation and hyper-responsiveness [32, 33]. The expression of IGFBP-3 is frequently decreased in tumors and this decrease is often a consequence of promoter methylation. IGFBP-3 has CpG islands in its promoters [34]. In our exploratory experiment, expression of IGFBP-3 was increased significantly in MBD2 knockout Jurkat T cells (Additional file 1).

## Conclusions

In summary, a neutrophilic inflammatory phenotype of asthma model was established successfully. Our study suggested that MBD2 regulates Th17 cell differentiation and IL-17 expression in neutrophils-dominant asthma through HIF-1α. Our findings uncover new roles for HIF-1α and MBD2, and provide novel insights into the epigenetic regulation of neutrophils-dominant asthma.

## Additional file

Additional file 1: IGFBP3 expression in Jurkat T cells with MBD2 gene knockout. (DOC 58 kb)

## Abbreviations

BALF: Bronchoalveolar lavage fluid
GATA3: GATA-binding protein 3
HDM: House dust mite
HIF-1α: Hypoxia inducible factor-1α
IL-17: Interleukin-l7
IL-4: Interleukin-4
LPS: Lipopolysaccharide
MBD2: Methtyl-CpG binding domain protein 2
Mch: Methacholine
OVA: Ovalbumin
RL: Lung resistance
RORγt: Orphan nuclear receptor
Th17: T helper 17 cells
Th2: T helper 2 cells

## References

[1] Robinson DS, Hamid Q, Ying S, Tsicopoulos A, Barkans J, Bentley AM (1992). Predominant TH2-like bronchoalveolar T-lymphocyte population in atopic asthma. N Engl J Med.

[2] Douwes J, Gibson P, Pekkanen J, Pearce N (2002). Non-eosinophilic asthma: importance and possible mechanisms. Thorax.

[3] Berry M, Morgan A, Shaw DE, Parker D, Green R, Brightling C (2007). Pathological features and inhaled corticosteroid response of eosinophilic and non-eosinophilic asthma. Thorax.

[4] Shannon J, Ernst P, Yamauchi Y, Olivenstein R, Lemiere C, Foley S (2008). Differences in airway cytokine profile in severe asthma compared to moderate asthma. Chest.

[5] Hastie AT, Moore WC, Meyers DA, Vestal PL, Li H, Peters SP (2010). Analyses of asthma severity phenotypes and inflammatory proteins in subjects stratified by sputum granulocytes. J Allergy Clin Immunol.

[6] Moore WC, Hastie AT, Li X, Li H, Busse WW, Jarjour NN (2014). Sputum neutrophil counts are associated with more severe asthma phenotypes using cluster analysis. J Allergy Clin Immunol.

[7] McKinley L, Alcorn JF, Peterson A, Dupont RB, Kapadia S, Logar A (2008). TH17 cells mediate steroid-resistant airway inflammation and airway hyperresponsiveness in mice. J Immunol (Baltimore, Md: 1950).

[8] Newcomb DC, Peebles RS (2013). Th17-mediated inflammation in asthma. Curr Opin Immunol.

[9] Walczak-Drzewiecka A, Salkowska A, Ratajewski M, Dastych J (2013). Epigenetic regulation of CD34 and HIF1A expression during the differentiation of human mast cells. Immunogenetics.

[10] Shi LZ, Wang R, Huang G, Vogel P, Neale G, Green DR (2011). HIF1alpha-dependent glycolytic pathway orchestrates a metabolic checkpoint for the differentiation of TH17 and Treg cells. J Exp Med.

[11] Kim SR, Lee KS, Park HS, Park SJ, Min KH, Moon H (2010). HIF-1α inhibition ameliorates an allergic airway disease via VEGF suppression in bronchial epithelium. Eur J Immunol.

[12] Esposito S, Tenconi R, Lelii M, Preti V, Nazzari E, Consolo S (2014). Possible molecular mechanisms linking air pollution and asthma in children. BMC Pulm Med.

[13] Collison A, Siegle JS, Hansbro NG, Kwok CT, Herbert C, Mattes J (2013). Epigenetic changes associated with disease progression in a mouse model of childhood allergic asthma. Dis Model Mech.

[14] Berger J, Bird A (2005). Role of MBD2 in gene regulation and tumorigenesis. Biochem Soc Trans.

[15] Daan de Boer J, Roelofs JJ, de Vos AF, de Beer R, Schouten M, Hommes TJ (2013). Lipopolysaccharide inhibits Th2 lung inflammation induced by house dust mite allergens in mice. Am J Respir Cell Mol Biol.

[16] Hoffman SM, Tully JE, Nolin JD, Lahue KG, Goldman DH, Daphtary N (2013). Endoplasmic reticulum stress mediates house dust mite-induced airway epithelial apoptosis and fibrosis. Respir Res.

[17] Locke NR, Royce SG, Wainewright JS, Samuel CS, Tang ML (2007). Comparison of airway remodeling in acute, subacute, and chronic models of allergic airways disease. Am J Respir Cell Mol Biol.

[18] Wang YH, Voo KS, Liu B, Chen CY, Uygungil B, Spoede W (2010). A novel subset of CD4(+) T(H)2 memory/effector cells that produce inflammatory IL-17 cytokine and promote the exacerbation of chronic allergic asthma. J Exp Med.

[19] Hu G, Tang Q, Sharma S, Yu F, Escobar TM, Muljo SA (2013). Expression and regulation of intergenic long noncoding RNAs during T cell development and differentiation. Nat Immunol.

[20] Vlahos R, Bozinovski S, Jones JE, Powell J, Gras J, Lilja A (2006). Differential protease, innate immunity, and NF-kappaB induction profiles during lung inflammation induced by subchronic cigarette smoke exposure in mice. Am J Physiol Lung Cell Mol Physiol.

[21] Wenzel SE, Schwartz LB, Langmack EL, Halliday JL, Trudeau JB, Gibbs RL, Chu HW (1999). Evidence that severe asthma can be divided pathologically into two inflammatory subtypes with distinct physiologic and clinical characteristics. Am J Respir Crit Care Med.

[22] Alcorn JF, Crowe CR, Kolls JK (2010). TH17 cells in asthma and COPD. Annu Rev Physiol.

[23] Wan H, Winton HL, Soeller C, Tovey ER, Gruenert DC, Thompson PJ, Stewart GA, Taylor GW, Garrod DR, Cannell MB (1999). Der p 1 facilitates transepithelial allergen delivery by disruption of tight junctions. J Clin Invest.

[24] Michel O, Kips J, Duchateau J, Vertongen F, Robert L, Collet H, Pauwels R, Sergysels R (1996). Severity of asthma is related to endotoxin in house dust. Am J Respir Crit Care Med.

[25] Celedon JC, Milton DK, Ramsey CD, Litonjua AA, Ryan L, Platts-Mills TA, Gold DR (2007). Exposure to dust mite allergen and endotoxin in early life and asthma and atopy in childhood. J Allergy Clin Immunol.

[26] Jia A, Wang Y, Sun W, Xiao B, Lin M, Wei Y (2017). Comparison of the roles of house dust mite allergens, ovalbumin, and lipopolysaccharides in the sensitization of severe neutrophil asthma. Exp Ther Med.

[27] Hutchins AS, Artis D, Hendrich BD, Bird AP, Scott P, Reiner SL (2005). Cutting edge: a critical role for gene silencing in preventing excessive type 1 immunity. J Immunol.

[28] Hutchins AS, Mullen AC, Lee HW, Sykes KJ, High FA, Hendrich BD (2002). Gene silencing quantitatively controls the function of a developmental trans-activator. Mol Cell.

[29] Huerta-Yepez S, Baay-Guzman GJ, Bebenek IG, Hernandez-Pando R, Vega MI, Chi L (2011). Hypoxia inducible factor promotes murine allergic airway inflammation and is increased in asthma and rhinitis. Allergy.

[30] Lukashev D, Caldwell C, Ohta A, Chen P, Sitkovsky M (2001). Differential regulation of two alternatively spliced isoforms of hypoxia-inducible factor-1 alpha in activated T lymphocytes. J Biol Chem.

[31] Zhou H, Chen X, Zhang WM, Zhu LP, Cheng L (2012). HIF-1α inhibition reduces nasal inflammation in a murine allergic rhinitis model. PLoS One.

[32] Lee YC, Jogie-Brahim S, Lee DY, Han J, Harada A, Murphy LJ (2011). Insulin-like growth factor-binding protein-3 (IGFBP-3) blocks the effects of asthma by negatively regulating NF-κB signaling through IGFBP-3R-mediated activation of caspases. J Biol Chem.

[33] Kim SR, Lee KS, Lee KB, Lee YC (2012). Recombinant IGFBP-3 inhibits allergic lung inflammation, VEGF production, and vascular leak in a mouse model of asthma. Allergy.

[34] Fridman AL, Rosati R, Li Q, Tainsky MA (2007). Epigenetic and functional analysis of IGFBP3 and IGFBPrP1 in cellular immortalization. Biochem Biophys Res Commun.

