# Supplementary material for: MBD2 regulates differentiation and function of Th17 cells in neutrophils- dominant asthma via HIF-1α
Source: J Inflamm (Lond). 2018 Aug 20;15:15. doi: 10.1186/s12950-018-0191-x (PMC6102869; doi:10.1186/s12950-018-0191-x)
Supplement: Supplementary file 1 — IGFBP3 expression in Jurkat T cells with MBD2 gene knockout. (DOC 58 kb) [file 12950_2018_191_MOESM1_ESM.doc]

Additional file 1

Authors: Li Xu^a†^(M.D.), Wen-Jin Sun^b†^(M.D.), Xu-Dong Xiang^c*^( M.D., Ph.D.)

Extended experimental procedured

*Plasmid transduction*

For MBD2 shRNA study, Jurkat T cells were transfected with plasmid containing the MBD2 shRNA hairpin sequences as described. Briefly, Jurkat T cells were incubated with plasmid either for a MBD2 shRNA or a control shRNA. After washes, the cells were cultured under new medium after 6h of transduction. The cells were then harvested 2 days later for Western blotting.

*Western blotting*

Total proteins were prepared using RIPA lysis buffer supplemented with protease inhibitors. Western blot was carried out by probing membranes with the indicated primary antibodies followed by incubation with an HRP-conjugated secondary antibody. IGFBP-3 antibodies were applied in the same manner.


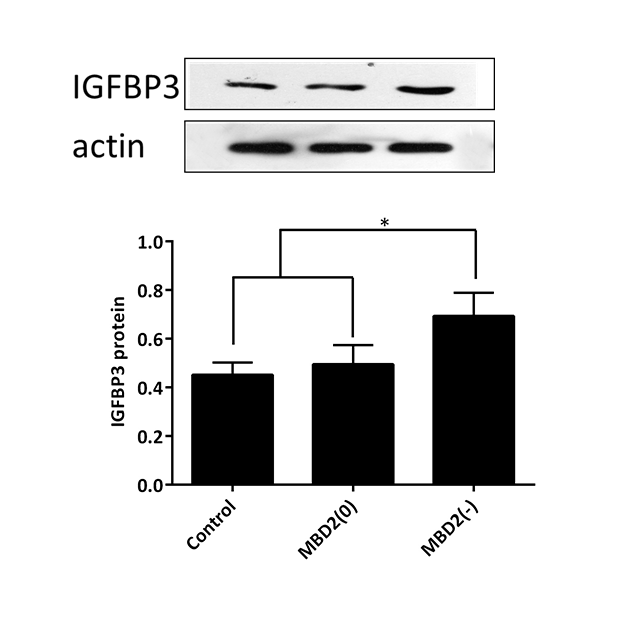


Fig. 8. IGFBP3 expression in Jurkat T cells with MBD2 gene knockout.

Jurkat T cells were transfected with an either MBD2-luciferase reporter plasmid or a luciferase containing empty plasmid. 48hrs post-transfection, cells were harvested and western blotting was carried out. *p<0.05 compared to other group. One-way ANOVA with Bonferroni's post hoc test were applied to analyze the results for significant differences (*p<0.05).
